# Screening and validation of key microRNAs regulating muscle development in Hanper sheep

**DOI:** 10.1371/journal.pone.0325054

**Published:** 2025-06-12

**Authors:** Yunxia Zhi, Boxin Hu, Shujun Tian, Ying Bai, Xiaoyong Chen

**Affiliations:** 1 College of Animal Science and Technology, Hebei Agricultural University, Baoding, China; 2 School of Life Science and Food Engineering, Hebei University of Engineering, Handan, China; University of Vermont College of Medicine, UNITED STATES OF AMERICA

## Abstract

**Background/Objectives:**

In sheep farming, the economic significance of meat characteristics is substantial, and advancing the genetic quality of livestock relies heavily on understanding the cellular mechanisms behind muscle growth and its regulation. This study examined miRNA expression patterns in the longissimus dorsi muscle tissue of Hanper sheep of various ages, with the goal of determining their biological functions and identifying miRNAs and their target mRNAs that influence muscle development and meat quality.

**Methods:**

Using the Image-Pro Plus 6.0 program and HE and fluorescent staining procedures, we measured the diameter of muscle fibers in the longissimus dorsi of Hanper sheep at three distinct ages (1, 7, and 13 months) in order to calculate the average fiber size. For the analysis of muscle fiber area, one-way Analysis of Variance was conducted using Statistical Package for the Social Sciences 25.0, with Least Significant Difference tests applied afterward to compare the different groups. Transcriptome sequencing was conducted to identify miRNAs, and bioinformatics tools were applied to predict their target genes. GO and KEGG functional annotations were used to analyze the biological functions of these target genes. RT-qPCR was performed to validate the expression levels of differential expressed miRNAs.

**Results:**

Muscle fiber diameter and area increased progressively with age, as indicated by HE and fluorescence staining. Four novel miRNAs identified for the first time in sheep were among the 116 differential expressed miRNAs that were found. These miRNAs were found to be involved in key pathways such as TGF-β, mTOR, Wnt, and MAPK, which regulate muscle growth and development. It was determined that three new miRNA-mRNA pairs included *oar-miR-133*/*MSC*, *oar-miR-148a*/*FST*, and *oar-miR-410-3p*/*NIN* may be essential for muscle growth. RT-qPCR results confirmed the expression trends observed in the transcriptome sequencing data.

**Conclusions:**

Our knowledge of the fundamental molecular mechanisms underpinning muscle growth and development is improved by the discovery of new miRNAs and the target genes that correspond to them. These findings may serve as new breeding targets for improving meat quality in sheep.

## Introduction

One crucial factor that significantly affects the financial gain of raising meat sheep is the quality of the meat. Skeletal muscle growth and development, which is mainly divided into two periods during pregnancy and after delivery is closely related to the quality of meat in sheep and poultry [1,2]. The quantity of myofiber during pregnancy and the diameter and volume of myofiber during the postnatal phase have an effect on the development of skeletal muscle [3,4]. Muscle fiber type, area, density, and diameter are important indicators of their properties [5,6]. It has been demonstrated that whereas muscle sensitivity rises as muscle fiber area and diameter decrease, meat softness and tensile strength decreases as these parameters increase [7]. Muscle development and growth are greatly influenced by the kind of myofiber, not only its size and diameter [8,9]. Meat quality and quantity are directly influenced by myofibrils, the basic building components of muscles [10]. However, several transcription factors and signal transduction pathways impede skeletal muscle growth and development [11,12].

A class of important regulators known as microRNAs (miRNAs) influences the growth and development of animals [13]. This factor influences gene activity beyond transcription and is crucial for controlling the growth, upkeep, and performance of skeletal muscle tissue. By binding to target genes, these miRNAs regulate muscle cell growth, differentiation, and death through signaling pathways [14]. Skeletal muscle development, including myogenesis, is a complicated process in which miRNAs play an important role [15]. In recent years, research on skeletal muscle has increasingly concentrated on how miRNAs influence gene regulation throughout the development of muscle fibers. For example, via controlling the expression of target genes, *miR-133a/b*, *miR-1*, and *miR-206* contributed to myoblast differentiation and proliferation [16–18]. Sheep skeletal muscle satellite cells may proliferate and differentiate myogenically more effectively when *miR-192* is present [19]. Primary bovine myoblast development and proliferation may depend on *miR-885* [20]. In mice, muscular dysfunction is regulated by *miR-7* [21]. Pig skeletal muscle growth and development are regulated by *miR-423-5p* [22]. Discovering the miRNAs involved in muscle differentiation and growth enhances our understanding of their regulatory roles and biological functions.

Hanper sheep is a high-yield meat breed developed by crossbreeding Dorper sheep (sire) with Small-tailed Han sheep (dam). After 15 years of population selection, and pure breeding, the genetic traits of this population have become stable, and the breed has now been maintained through six to eight generations of pure breeding [2]. This breed combines the fast growth rate and high-quality meat of Dorper sheep with the strong adaptability and high reproductive performance of Small-tailed Han sheep. Notably, Hanper sheep exhibit excellent meat production performance and meat quality. At six months of age, Hanper sheep have an average carcass weight of 30.29 kg, a dressing percentage of 60.82%, a lean meat yield of 80.19%, a longissimus dorsi muscle area of 19.60 cm^2^, an average muscle fiber diameter of 27.28 μm, and a high marbling score, with intramuscular fat content in the *longissimus dorsi* reaching 18.46% [23]. Hanper sheep possess typical meat sheep characteristics, with uniform body conformation, stable genetic traits, and strong adaptability. They are suitable for both pure breeding and crossbreeding to utilize heterosis for meat production and are well-adapted to the regional characteristics, climatic conditions, and natural environment of the North China Plain farming areas. However, as a newly developed meat sheep breed, the molecular regulatory mechanisms underlying muscle growth and development in Hanper sheep remain unclear. Investigating how molecular factors influence muscle formation and growth at different developmental stages is crucial for optimizing breeding strategies and improving the production efficiency of meat sheep. Therefore, this study employed transcriptome sequencing to analyze the longissimus dorsi muscle of Hanper sheep at different developmental stages (1 month, 7 months, and 13 months old), aiming to identify miRNAs associated with skeletal muscle development.

## Materials and methods

### Sample collection

This study was conducted with authorization from the Animal Welfare and Ethics Committee of the Institute of Animal Science, Hebei Agricultural University. All methods and procedures involving experimental animals were carried out in accordance with the relevant guidelines (Document Approval No: 2020070). In this study, we selected three uncastrated Hanper rams at three different developmental stages—1 month (M1), 7 months (M7), and 13 months (M13) —from the Hebei Liansheng Agricultural Development Co., Ltd. sheep farm. At each stage, three rams with similar body weights, good health status, and no genetic kinship were chosen. To ensure animal welfare during transport, the rams underwent a pre-shipment quarantine in a separate area. Careful handling practices were implemented throughout the journey, avoiding excessive confinement and unnecessary force to mitigate both stress responses and injuries. After arriving at the slaughterhouse, the sheep were humanely slaughtered according to the “Livestock and Poultry Slaughtering Operation Regulations for Sheep” (NY/T 3469−2019). Following the butchering process, *longissimus dorsi* muscle specimens were promptly excised and subjected to rapid freezing using liquid nitrogen. To maintain sample integrity, they were later transferred to an ultra-low temperature environment of −80°C for preservation.

### Measurement of muscle fiber area

The methods of fluorescence staining and Hematoxylin and Eosin (HE) staining were applied. In accordance with Guo’s methodology, the *longissimus dorsi* muscle tissue was fixed in a 4% neutral paraformaldehyde solution before HE staining [1]. Briefly, 5μm-thick paraffin slices were created by removing the fixed muscle tissue. Fix a small piece of tissue with wax, cut it into thin slices with a slicer, then perform HE staining, staining steps are dewaxing, covering water, hematoxylin staining, 5% acetic acid differentiation, returning blue, eosin staining, dehydration, dropping neutral resin. The stained samples were viewed under a microscope with a 15 × 40 zoom, using an eyepiece micrometer for accurate measurements. Fifteen images were captured from diverse, brightly lit areas across different focal points. Measuring their diameter and calculating the average using Image-Pro Plus 6.0 software. Data related to the muscle fiber areas were processed with Statistical Package for the Social Sciences 25.0 software, employing a one-way ANOVA analysis to assess variance. Multiple comparisons were conducted using the Least Significant Difference (LSD) method to pinpoint any notable differences between the groups.

### Total RNA extraction and miRNA sequencing

Following the comprehensive directions for the process, the Trizol reagent Kit (Invitrogen, Carlsbad, California (CA), United States of America (USA)) was used to extract RNA from *longissimus dorsi* muscle tissue. RNA degradation and contamination were assessed using 1% agarose gel electrophoresis. RNA purity was determined with a NanoPhotometer® spectrophotometer (IMPLEN, CA, USA). RNA concentration was measured using the Qubit® RNA Assay Kit on a Qubit® 2.0 Fluorometer (Life Technologies, CA, USA). RNA integrity was evaluated with the RNA Nano 6000 Assay Kit on the Agilent Bioanalyzer 2100 system (Agilent Technologies, CA, USA). For each sample, 3 μg of total RNA was used as the starting material for small RNA library construction. Libraries were prepared using the NEBNext® Multiplex Small RNA Library Prep Set for Illumina® (NEB, USA.), following the manufacturer’s recommended protocol, with index sequences added to distinguish between different samples. Finally, library quality was assessed on the Agilent Bioanalyzer 2100 system using High Sensitivity DNA Chips. Clustering of the index coded samples was performed on a cBot Cluster Generation System using the TruSeq SR Cluster Kit v3-cBot-HS (Illumina), according to the manufacturer’s instructions. After cluster generation, sequencing of the prepared libraries was carried out on the Illumina HiSeq 2500 platform, generating 50 bp single-end reads.

### Data processing

From the clean reads that passed quality control, we extracted sequences that fell within a certain length range. The alignment of short RNA tags to the reference genome (*Ovis aries Oar_v4.0*) was done using Bowtie (bowtie-0.12.9) [24], ensuring that no mismatches were tolerated. The small RNA sequences mapped to the genome were used for the identification of known miRNAs. MiRBase 20.0 was used as the reference database, and miRNA prediction along with secondary structure visualization was performed using Mirdeep2 (mirdeep2_0_0_5) [24] and SR-tools-cli. The first nucleotide bias of miRNAs with specific lengths and the base preference at each position of all miRNAs were analyzed. To eliminate small RNA tags originating from protein-coding genes, repetitive sequences, rRNA, tRNA, snRNA, and snoRNA, the small RNA tags were aligned to the RepeatMasker (open-4.0.3) database, Rfam database, or species-specific datasets. The hairpin structure of miRNA precursors was used to predict novel miRNAs. In this study, a combination of MiREvo (MiREvo_v1.1) [25] and Mirdeep2 [24] was used to predict unannotated small RNA tags by analyzing secondary structures, Dicer endoribonuclease (Dicer) cleavage sites, and minimum free energy (MFE). Custom scripts were used to calculate the counts of the identified miRNAs, and both the first nucleotide bias for miRNAs of specific lengths and the nucleotide preference at each position for all miRNAs were analyzed separately. Target gene prediction was conducted using miRanda(miRanda-3.3a) [26]. The expression levels of miRNAs were normalized and estimated using Transcripts Per Million (TPM), according to the following formula [27]: Normalized expression = mapped readcounts/Total reads*1, 000, 000.

### Differential expression miRNA analyses and functional pathway enrichment

The expression levels of miRNAs were normalized using TPM. Differentially expressed miRNAs (DEMs) between the *longissimus dorsi* muscle tissues of Hanper sheep at 1 month, 7 months, and 13 months were identified using the Differential Expression Sequencing R package (version 1.8.3). P-values were adjusted for multiple hypothesis testing using the Benjamini & Hochberg method to control the False Discovery Rate (*FDR*). By default, an adjusted *P-value (FDR)* < 0.05 was considered the threshold for significant differential expression. The criteria for selecting DEMs were as follows: *TPM* > 1, *|log2(FC)| *≥ 1, *P-value or FDR *< 0.05, and at least three biological replicates [28].

Gene Ontology (GO) enrichment analysis was performed on the predicted target genes of the DEMs. The Goseq (Release 2.12) program, which is based on the Wallenius non-central hypergeometric distribution [30], was used to investigate GO enrichment. The significance thresholds for GO enrichment were set at *P* < 0.05 or *FDR* < 0.05. Kyoto Encyclopedia of Genes and Genomes (KEGG) enrichment analysis was conducted using the KOBAS (v2.0) software [29,30] to evaluate the statistical enrichment of candidate target genes in KEGG pathways and identify important biological pathways potentially regulated by miRNAs. The significance thresholds for KEGG enrichment were *P* < 0.05 or *FDR* < 0.05. Target gene prediction was performed through cross-validation using multiple databases, including miRanda and TargetScan, and further filtered by RNA sequencing data to remove genes with low expression. Additionally, to explore the interactions between miRNAs and muscle growth related genes, an interaction network diagram was constructed using Cytoscape software, revealing regulatory relationships at the molecular level.

### Reverse Transcription quantitative Polymerase Chain Reaction (RT-qPCR) verification

RNA isolated from the *longissimus dorsi* muscle was processed for reverse transcription with the M5 miRNA cDNA Synthesis Kit, a tool from Mei5 Biotechnology Co., Ltd, specializing in small RNA first strand synthesis. Using U6 Small Nuclear RNA (*U6*) as an internal reference. Table 1 presents the primer specifications. The quantitative PCR process was conducted following the protocols of the M5 miRNA qPCR Assay Kit, a system designed for miRNA detection using fluorescent methods (Mei5 Biotechnology Co., Ltd). The reaction system was as follows: 10 μL 2 × M5 miRNA qPCR Mix, 7.2 uL of RNase-free water, 2 μL of cDNA template, 0.4 μL of forward primer, and 0.4 μL of reverse primer. All qPCR reactions were conducted using a real-time PCR equipment from Thermo Fisher Scientific, an ABI Prism 7500, located in Waltham, MA, USA. To determine the target gene’s expression, the relative quantification approach of *2*^*-∆∆Ct*^ was used.

**Table 1:** Primer sequences utilized in quantitative fluorescence PCR.

Primer	Primer sequence (5’to3’)	Length, nt
oar-miR-133	TTGGTCCCCTTCAACCAGCTGT	22
oar-miR-148a	TCAGTGCACTACAGAACTTTGT	22
oar-miR-191	CAACGGAATCCCAAAAGCAGCT	22
oar-miR-27a	TTCACAGTGGCTAAGTTCCGC	21
oar-miR-30a-5p	TGTAAACATCCTCGACTGGAAGC	23
oar-miR-3957-3p	ACGCACAGCACCTCACTGAGCT	22
oar-miR-410-3p	AATATAACACAGATGGCCTGT	21
oar-miR-541-5p	AAAGGATTCTGCTGTCGGTCCCACT	25
U6	AGTGCAGGGTCCGAGGTATT	20

## Results

### Measurement of muscle fiber area

**Fig 1:**
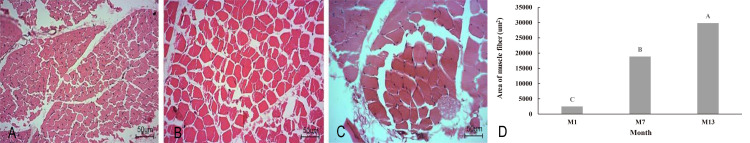
HE staining and area of muscle fiber at the different age stages of Hanper sheep. (A) 1 month- old. (B) 7-month-old. (C) 13-month-old. (D) Area of muscle fiber of different months old sheep. To indicate significant differences, distinct uppercase letters were employed (*P* < 0.01), whereas the same letter suggested the lack of any significant distinction (*P* > 0.05).

The *longissimus dorsi* muscle of histology structure was conducted by HE staining. The muscle fiber diameter of 1 month-old (Fig 1A) was smaller than those of 7-month-old (Fig 1B) and 13-month-old sheep (Fig 1C). The data indicated that the muscle fiber diameter was increased with month old. Moreover, the area of muscle fiber in different month-old sheep were measured. Our data revealed a substantial growth in the *longissimus dorsi* muscle fibers, from 2490 μm^2^ at one month of age to 18850 μm^2^ at seven months, further escalating to 29840 μm^2^ by thirteen months, with statistical significance (*P* < 0.01). (Fig 1D and Table 2), indicating that the muscle fiber area increased with the increase of age.

**Table 2:** Determination of meat quality indexes.

Month	pH_24_	cooking holding percentage (%)	water loss rate (%)	shear force (N)	diameter of muscle fiber (μm^2^)
1(M1)	5.53 ± 0.05^B^	52.56 ± 3.12	36.9 ± 0.60^B^	2.46 ± 0.05	2490^A^
7(M7)	5.49 ± 0.07^B^	51.70 ± 0.79	43.7 ± 3.37^A^	3.19 ± 0.26	18850^B^
13(M13)	6.05 ± 0.09^A^	49.20 ± 4.30	46.2 ± 2.92^A^	4.03 ± 1.36	29840^C^

Note: Different letters indicated that the difference was extremely significant (*P *< 0.01), and the same letter indicated that the difference was not significant (*P* > 0.05).

### Sequencing data quality and statistics

A total of 163,909,364 raw reads were obtained from nine samples of Hanper sheep’s longissimus dorsi muscle tissue at M1, M7, and M13. After quality control, 162,375,451 high-quality clean reads were obtained, accounting for 99.06% of raw reads. These clean reads had *Q30* values between 98.77% and 99.20%, with a GC content of about 45%. With an error rate of under 0.01% (Table 3), the sequencing results in this research showed excellent accuracy in base identification, reflecting the reliability of the data generated. The length range of small RNA was from 18 to 40 nucleotides (*nt*), most of which were between 21 and 23 nt (about 90.31%). Among them, the length distribution ratio of 22 nt was the highest (61.39%−67.86%), followed by 23 nt (15.99%−21.24%) and then by 21 nt (6.61%−10.77%).

**Table 3:** Quality processing of sequencing data and genome comparison results of reads.

Sample name	Total sRNA	Mapped sRNA	“+”Mapped sRNA	“_”Mapped sRNA	Raw reads	Clean reads	Error rate	Q30	GC content	Clean bases
M1_1	17119350	16090529 (93.99%)	12881932 (75.25%)	3208597 (18.74%)	17473013	17368993 (99.40%)	0.01%	99.15%	45.54%	0.874G
M1_2	18658326	17518711 (93.89%)	14006221 (75.07%)	3512490 (18.83%)	19093812	18966983 (99.34%)	0.01%	99.08%	45.52%	0.955G
M1_3	18828007	17648875 (93.74%)	14207157 (75.46%)	3441718 (18.28%)	19484782	19221586 (98.65%)	0.01%	99.00%	46.00%	0.974G
M7_1	18902372	18137147 (95.95%)	15061077 (79.68%)	3076070 (16.27%)	19376465	19159719 (98.88%)	0.01%	98.95%	44.01%	0.969G
M7_2	17143053	16409031 (95.72%)	13671710 (79.75%)	2737321 (15.97%)	17693345	17508199 (98.95%)	0.01%	99.20%	44.30%	0.885G
M7_3	16219534	15462332 (95.33%)	12797447 (78.90%)	2664885 (16.43%)	16547765	16413896 (99.19%)	0.01%	99.08%	44.15%	0.827G
M13_1	16984371	16269950 (95.79%)	13333251 (78.50%)	2936699 (17.29%)	17378541	17226973 (99.13%)	0.01%	98.77%	44.25%	0.869G
M13_2	16988614	16348734 (96.23%)	13347660 (78.57%)	3001074 (17.67%)	17380155	17182736 (98.86%)	0.01%	99.05%	43.91%	0.869G
M13_3	19154973	18490625 (96.53%)	15542869 (81.14%)	2947756 (15.39%)	19481486	19326366 (99.20%)	0.01%	99.10%	43.87%	0.974G

### miRNAs prediction and DEMs identification

All clean reads from nine samples were aligned to the whole genome, and the results showed that the proportion of reads matching the reference sequence ranged from 93.74% to 96.53%, with positive strand matching accounting for 75.07% to 81.14% and negative strand matching accounting for 15.39% to 18.83% (Table 3). Among all types of sRNA, miRNA accounted for only 1.15%−1.32%. However, in the number statistics of all types of sRNA, exon accounted for the highest proportion (45.6%−62.77%), followed by other (19.73%−21.9%), and then miRNA (9.21%−18.6%). After analyzing miRNA expression differences among various groups, it was observed that nearly half (47.56%) of the miRNAs, with *TPM* levels higher than 60, were present in the muscle samples from Hanper sheep at three separate growth stages.

**Fig 2:**
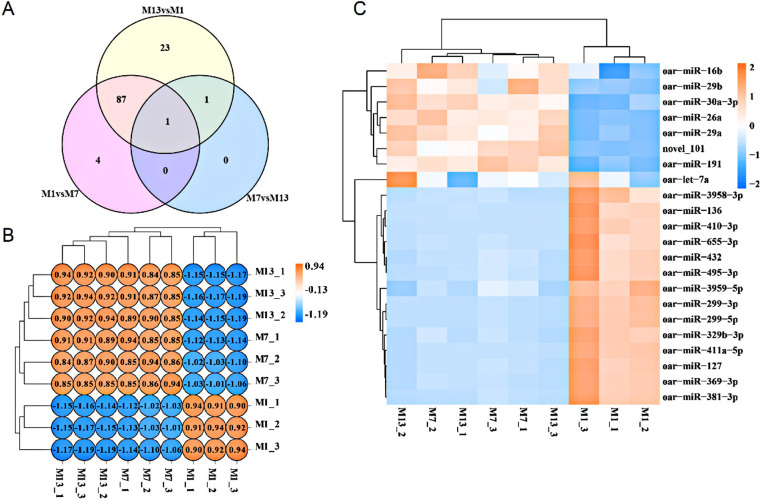
miRNA prediction and DEMs identification. (A) Venn diagram illustrating differential expression of miRNAs across distinct developmental stages. (B) Correlation heatmap depicting relationships among the nine samples. (C) Hierarchical clustering heatmap presenting the top 15 DEMs included 22 miRNAs.

As a result, a total of 116 miRNAs included 112 known mature miRNAs, 4 new candidate miRNAs (*novel_101*, *novel_256*, *novel_297*, *novel_340*) in nine samples were identified. Among them, 11 miRNAs did not have predicted target genes. The 116 differential expressed miRNAs (S1 Table; Fig 2A) in the *longissimus dorsi* muscle of Hanper sheep at different growth stages were analyzed. Among them, target genes for 11 miRNAs were not predicted, while target genes for the remaining 105 differential expressed miRNAs were predicted, resulting in a total of 3970 target genes (S2 Table). To eliminate errors caused by abnormal samples, the Pearson correlation between evaluation samples was calculated to be *R*^*2*^ > 0.9 (Fig 2B). Between M1 and M7, 92 miRNAs were found to have differential expression, 48 of which were up-regulated and 44 of which were down-regulated. The top 10 DEMs with the largest fold change were including *oar-miR-410-3p*, *oar-miR-136*, *novel_101*, *oar-miR-411a-5p*, *oar-miR-299-5p*, *oar-miR-29a*, *oar-miR-655-3p*, *oar-miR-299-3p*, *oar-miR-26a*, *oar-miR-29b*. There were 112 differential expressed miRNAs between the ages of M1 and M13, with 62 up-regulated and 50 down-regulated. The top 10 DEMs with the largest fold change included *miR-127*, *miR-369-3p*, *miR-136*, *miR-655-3p*, *miR-411a-5p*, *miR-410-3p*, *miR-495-3p*, *miR-3958-3p*, *miR-381-3p*, and *miR-329b-3p*. Between the M7 and M13 stages, just two miRNAs exhibited significant expression differences: *oar-miR-381-3p* and *oar-miR-3959-5p*, both showing increased expression levels. Based on the evaluation of fold change and adjusted significance level, 116 DEMs were screened with the criteria of *padj* < 0.05. The top 15 (or all) miRNAs in each stage expression level included 22 different miRNAs (S3 Table). Using cluster analysis, we identified three separate gene groups from the longitudinal data, with distinct patterns of up-regulation and down-regulation observed (Fig 2C). Furthermore, we predicted target genes for most of the DEMs which associated with regulation of muscle growth and development in previous study such as *oar-miR-27a*/*NOXA1*, *oar-30a-5p*/*CDK15*, *oar-miR-191*/ Ribosomal Protein S18(*RPS18*), *oar-miR-541-5p*/*TPM2*, *oar-miR-3957-3p*/*DOCK1* and miRNA-mRNA pairs included *oar-miR-133*/ Mesenchymal Stem Cells (*MSC*), *oar-miR-148a*/*FST* and *oar-miR-410-3p*/*NIN*.

### GO term and KEGG pathway enrichment

**Fig 3:**
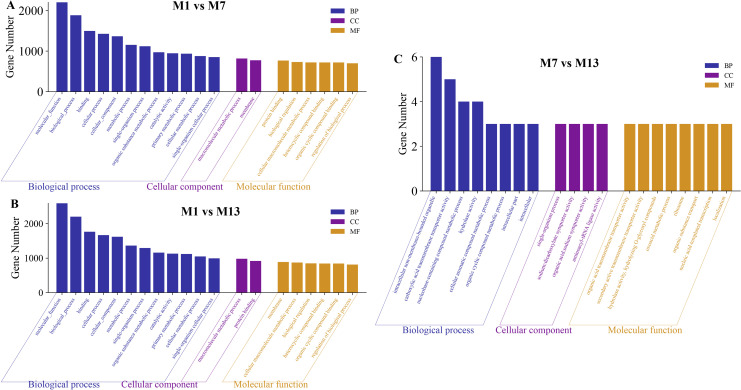
Functional enrichment analysis using GO. (A) M1vsM7. (B) M1vsM13. (C)M7vsM13.

**Fig 4:**
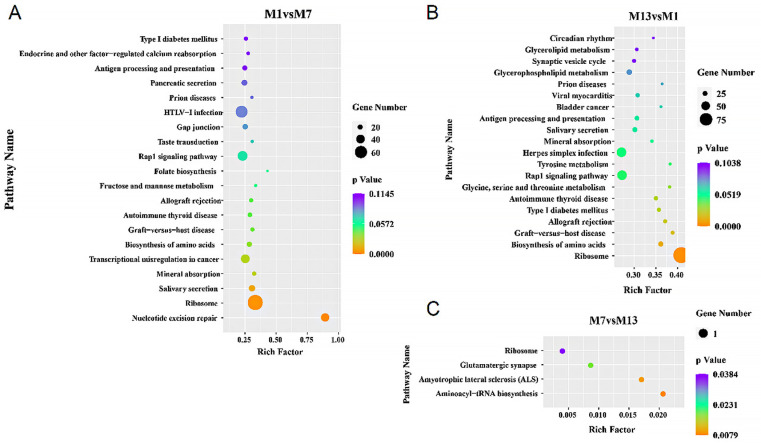
Enrichment analyses of KEGG pathways. (A) M1vsM7. (B) M13vsM1. (C)M7vsM13.

In the comparison between M1 and M7, GO enrichment analysis identified a substantial (*P *< 0.05) concentration of 89,309 target genes distributed over 3,248 functional groups (S4 Table). The biological process, cellular component, and molecular function categories were over represented in the top 20 DEMs including target genes (Fig 3A). 107213 target genes of M1 vs M13 comparison were enriched in 3456 GO terms (S4 Table). The top 20 DEMs with target genes were enriched in biological process, cellular component, and molecular function (Fig 3B). There were 159 GO terms enriched with 232 target genes in the M7 vs M13 group (S4 Table). Among the top 20 DEMs, the involved target genes were linked to various biological processes, including non-membrane-bounded organelles within cells, activities of carboxylic acid transporters, and cellular components like ribosomes. They were also related to molecular functions in the metabolism of aromatic compounds within cells (Fig 3C). The results revealed that host genes of miRNA were mainly involved in functions of muscle growth and development, such as muscle growth and development (*GO:0003012, GO:0090257*), muscle contraction regulation (*GO:0006937, GO:0006940*), muscle structure development (*GO:0061061*), and muscle organ development (*GO:0007517*). Moreover, these target genes were enriched to 272 KEGG pathways, of which the most significant pathways were muscle development related pathways including Transforming Growth Factor Beta (TGF-β) (04350), mammalian Target of Rapamycin (mTOR) (04150), Wnt (04310), and Mitogen-Activated Protein Kinase (MAPK) (04010) (S5 Table). The top 20 KEGG-enriched pathways for differential miRNAs in M1 vs M7 primarily involved in the ribosome, nucleotide excision repair, Rap1 signaling pathway, HTLV-I infection, and transcriptional misregistration in cancer (Fig 4A). The top 20 DEMs in the M1 vs M13 comparison revealed enrichment in several key pathways, such as Ribosomal function, Rap1 signaling, and the infection process of herpes simplex (Fig 4B). There were aminoacyl-tRNA biosynthesis, amyotrophic lateral sclerosis (ALS), glutamate synapse, and ribosome enriched in the top 20 KEGG pathways of M7 vs M13 group (Fig 4C).

### MiRNA-mRNA regulatory network analysis

**Fig 5:**
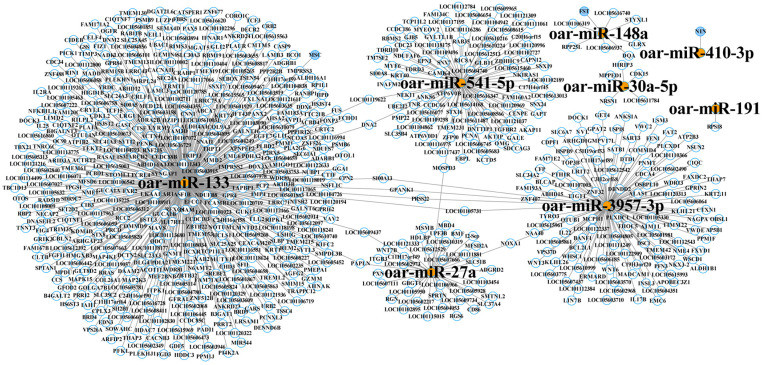
Network depicting interactions between miRNAs and mRNAs.

Eight miRNAs, including *oar-miR-27a*/*NOXA1*, *oar-30a-5p*/*CDK15*, *oar-miR-191*/*RPS18*, *oar-miR-541-5p*/*TPM2*, *oar-miR-3957-3p*/*DOCK1*, *oar-miR-133*/*MSC*, *oar-miR-148a*/*FST* and *oar-miR-410-3p*/*NIN* were thought to be connected to muscle growth based on function annotation and enrichment analyses. A comprehensive network model was developed to illustrate the interaction among eight specific miRNAs and their corresponding target genes, incorporating 703 mRNAs and forming 699 distinct associations (Fig 5). Analysis of the interaction network highlighted the significant involvement of *oar-miR-133*, *oar-miR-27a*, *oar-miR-3957-3p*, and *oar-miR-541-5p* with a large proportion of target genes, implying their essential contribution to the molecular mechanisms underlying muscle growth in sheep.

### Verification of target genes

**Fig 6:**
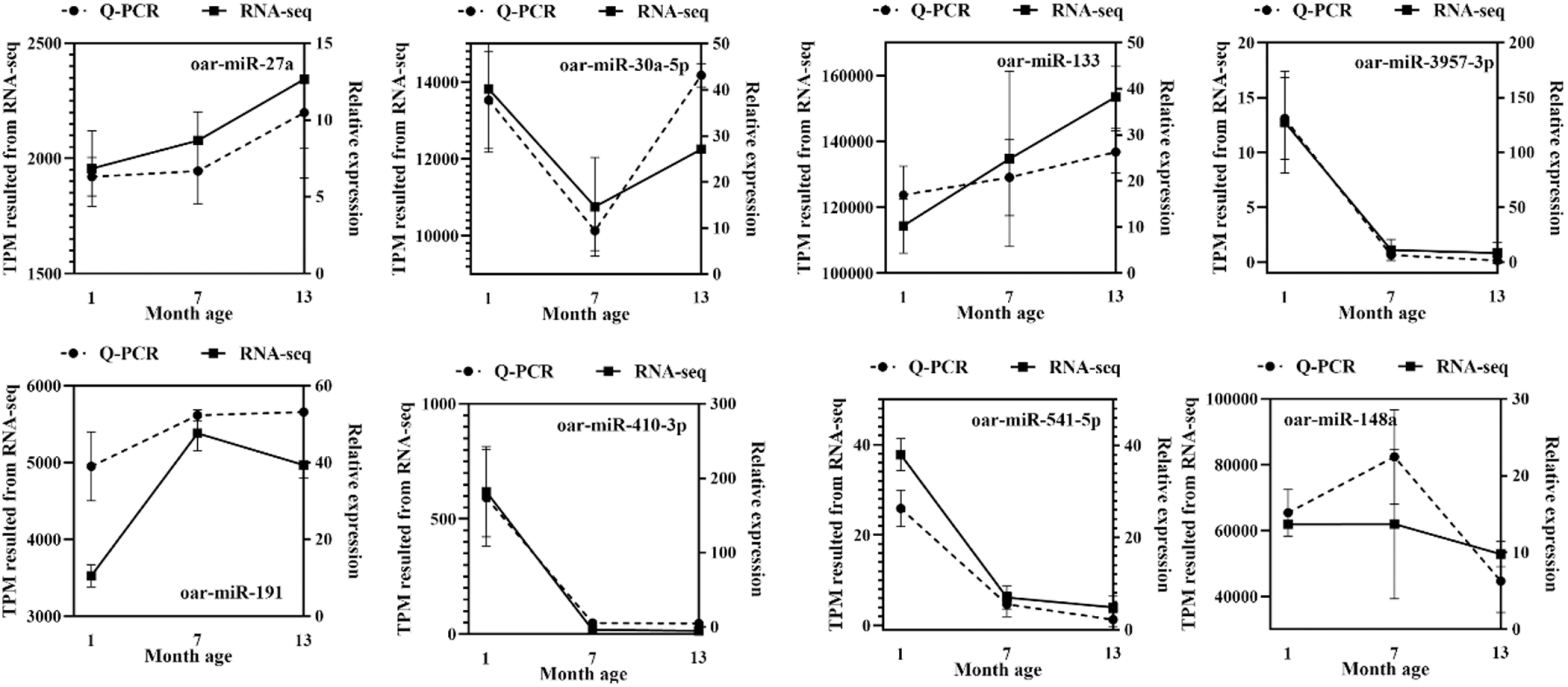
Experimental and sequencing validation of the 8 miRNAs.

By RT-qPCR, the selected eight miRNAs related to muscles in Hanper sheep at different ages, including *miR-133*, *miR-148a*, *miR-191*, *miR-27a*, *miR-30a-5p*, *miR-3957-3p*, *miR-410-3p*, and *miR-541-5p*, were also used to verify the accuracy and reliability of the sequencing. A comparison with RNA sequencing analysis revealed a highly similar trend, supporting the notion that the sequencing methodology precisely captured and reliably reflected the expression profiles of miRNAs linked to muscle development in Hanper sheep (Fig 6).

## Discussion

The most fundamental unit of muscle is muscle fiber, and the quality and performance of meat production in animals are directly impacted by its properties [5,6]. Sheep develop muscle fibers before birth, particularly during mid-gestation [31]. On the other hand, their postnatal muscular development is mostly reliant on muscle fiber hypertrophy [32]. Between birth and six months, a lamb grows incredibly quickly. Their skeletal muscle fibers have matured in size after growing for 1 to 1.5 years [33]. Therefore, we conducted a fibers diameter and area comparison analysis of Hanper sheep *longissimus dorsi* muscle at 1, 7 and 13 months of age. A clear age-related trend was observed, with younger lambs at one month displaying a reduced muscle fiber area relative to their counterparts at seven and thirteen months, implying a progressive enlargement of muscle fibers over time.

MiRNAs regulate gene expression by binding to the 3’ untranslated regions of target genes, leading to mRNA degradation or transnational repression [11]. Through functional annotation and enrichment analysis, this study identified several DEMs closely associated with muscle growth, including *oar-miR-191*, *oar-miR-27a*, *oar-miR-30a-5p*, *oar-miR-3957-3p*, and *oar-miR-541-5p*. These miRNAs may play a role in the formation, maintenance, and metabolism of skeletal muscle by regulating key signaling pathways and target genes. Among them, *miR-27a* is a critical miRNA that promotes myoblast proliferation and differentiation by targeting and suppressing myostatic [34]. It also participates in muscle fibre type transformation, such as inhibiting *MYH7* and regulating *PGC-1α*-associated mitochondrial metabolism genes [35,36]. Under pathological conditions, *miR-27a* alleviates muscle atrophy in chronic kidney disease or exercise intervention through the Akt/FoxO1 signaling pathway [37,38]. Additionally, it is associated with miR-27a secreted by adipose tissue, mediating insulin resistance [39]. Moreover, *miR-27a* facilitates osteogenic differentiation via exosomes and has potential roles in cardioprotection and anti-fibrosis [40–42]. *MiR-30a-5p* inhibits myocyte differentiation by targeting *MBNL1/2*, thereby regulating the alternative splicing of *Trim55* and *INSR*, which in turn affects muscle signaling pathways. In cattle, *miR-30a-5p* represses the expression of differentiation markers *MHC* and *MyoG* [43], which were linked to adipogenesis in buffalo muscle [44]. Under pathological conditions, *miR-30a-5p* suppresses autophagy by targeting *ATG5*, thereby promoting vascular smooth muscle cell proliferation and migration, exacerbating venous graft restenosis [45]. It also regulates vascular smooth muscle cell phenotypical transformation through the *SMAD1*/TGF-β axis, contributing to atherosclerosis [46]. In myocardial ischaemia-reperfusion injury, *miR-30a-5p* exacerbates myocardial damage through the *circ_0002612*/*miR-30a-5p/Ppargc1a/NLRP3* axis, while its inhibition can enhance mitochondrial function by activating *SIRT1* [47–49]. In viral myocarditis, *miR-30a-5p* modulates macrophage polarisation to promote inflammatory responses [50]. Furthermore, the *miR-30* family exhibits dysregulated expression in muscular dystrophy and skeletal muscle atrophy, participating in muscle metabolism regulation [51]. MiR-191-5p is positively correlated with muscle strength and muscle cross-sectional area, potentially enhancing muscle function via the mTOR pathway [52]. During skeletal muscle regeneration, it influences the proliferation and differentiation of muscle satellite cells by regulating p27 [53]. Changes in plasma *miR-191* levels following exercise suggest its responsiveness to metabolic and inflammatory signals [54]. In the skeletal muscle of low residual feed intake pigs, *miR-191* down regulation affects mitochondrial energy metabolism through the AMPK-PGC-1α pathway [55]. In vascular smooth muscle cells, *miR-191-5p* targets *PLCD1* to regulate apoptosis and inflammation, contributing to the progression of abdominal aortic aneurysms [53]. In a myocardial ischaemia model, *miR-191* influences cardiomyocyte survival via the TRAF3/Bcl-2 pathway [56]. Additionally, *miR-191* serves as a stable reference gene in expression studies [57]. *MiR-541-5p* has primarily been studied in the context of smooth muscle and fibrosis. In the Radial Ischemia Preconditioning Fibrosis model, it inhibits epithelial-mesenchymal transition and alleviates fibrosis by targeting *ZIC1* [58,59]. Furthermore, it regulates the PDE1A pathway in pulmonary fibroblasts, contributing to the fibrotic process [60]. Although no direct studies have linked *miR-541-5p* to skeletal muscle, its target gene *TPM2* encodes β-tropomyosin, which regulates actin-myosin interactions and is a crucial component of sarcomeric filaments [61]. *TPM2* mutations are associated with various congenital myopathies, including nemaline myopathy, cap myopathy, and distal arthrogryposis, with underlying mechanisms involving calcium sensitivity abnormalities and sarcomere structural disruption [62–65]. Research on *miR-3957-3p* is relatively limited, however, its predicted target gene *DOCK1* promotes myoblast fusion via Rac GTPase signaling [66,67] and cooperates with *ELMO* and *Crk/Crkl* in myogenesis [68,69]. In cardiomyocytes, *DOCK1* exerts anti-apoptotic effects through the AKT pathway [70], while *circ_DOCK1* regulates vascular smooth muscle cell proliferation and apoptosis [71]. In summary, these miRNAs participate in skeletal muscle development, regeneration, and disease processes through distinct signaling pathways and target genes. Future research should focus on elucidating their precise mechanisms in muscle growth, regeneration, atrophy, and related diseases, exploring their potential applications in clinical therapy.

Through DEM analysis and GO/KEGG pathway enrichment analysis, this study identified three novel miRNA-mRNA interaction pairs: *oar-miR-133*/*MSC*, *oar-miR-148a*/*FST*, and *oar-miR-410-3p*/*NIN*. *MiR-133* is a classic muscle-specific miRNA, primarily expressed in the heart and skeletal muscle, and consists of two subtypes: *miR-133a* and *miR-133b* [72]. By encouraging myoblast proliferation and controlling muscle fiber development by specifically inhibiting genes like serum response factor (*SRF*) and histone deacetylase 4 (*HDAC4*), it serves a dual regulatory function in myogenesis [75]. Additionally, it forms a feedback loop with the ERK1/2 signaling pathway to fine-tune muscle development [73]. Aberrant expression of *miR-133* is closely associated with myocardial hypertrophy, heart failure, and muscular dystrophy [74]. It also regulates muscle regeneration and fibrosis by targeting RhoA and interacting with lncRNAs (such as *Lnc133*). Exogenous *miR-133*, in combination with other miRNAs, has been shown to promote the regeneration of damaged muscles [75]. Its expression is directly regulated by *MEF2* and evolutionarily originates from chordates [76,77], highlighting its potential in muscle disease diagnosis and treatment [78,79]. Mesenchymal stem cells (*MSCs*) play a crucial role in muscle regeneration and disease treatment by secreting paracrine factors (such as exosomes), promoting myogenesis and angiogenesis, and reducing fibrosis [80]. *MSCs* derived from bone marrow, umbilical cord, and muscle have been shown to improve muscle function in Duchenne muscular dystrophy (*DMD*) mouse models [81], regulate the inflammatory microenvironment, and repair genetic myopathies by modulating *COL6* expression [82]. *MSCs* can also delay sarcopenia progression by activating satellite cells and improving mitochondrial function [83]. The application of novel delivery systems, such as sulfated alginate hydrogel-encapsulated *MSC* spheroids, has further enhanced muscle regeneration and reduced fibrosis deposition. Muscle-derived *MSCs* exhibit superior myogenic and osteogenic differentiation compared to bone-derived *MSCs*, suggesting their potential in autologous therapy [84]. Additionally, *MSCs* promote muscle repair by regulating the interaction between Treg cells and IL-33 [85]. This study is the first to reveal the targeting regulation between *oar-miR-133* and *MSCs*, indicating their synergistic role in muscle development and repair. The miR-133/*MSC* axis shows potential in muscle disease treatment, warranting further exploration of its molecular mechanisms and clinical applications. *MiR-410-3p* exhibits complex regulatory roles in various diseases, particularly in muscle, cardiovascular, and lipid metabolism disorders. Its circulating levels are elevated in *DMD* patients, suggesting its involvement in muscle pathology and its potential as a biomarker. In the cardiovascular system, *miR-410-3p* promotes Ang II-induced myocardial hypertrophy by inhibiting *Smad7* [86]. It is also upregulated in endothelial progenitor cells of coronary artery disease (*CAD*) patients, where it jointly suppresses *VEGFR2* expression, affecting blood flow recovery in ischemic tissues and providing a potential therapeutic target for *CAD* [87]. Furthermore, *miR-410-3p* inhibits *IRS-1*, blocking adipocyte differentiation and regulating lipid metabolism, and may contribute to fat loss in cancer-associated cachexia [88]. Notably, the function of this miRNA is disease-specific, and its regulatory role may vary across different diseases [86]. Ninein (*NIN*) is a microtubule-organizing regulator involved in microtubule reorganization and inflammation regulation in the muscular system. During skeletal muscle differentiation, *NIN* regulates nuclear envelope microtubule-organizing centers, influencing myotube formation [89]. In smooth muscle, it suppresses pro-inflammatory factor expression and alleviates inflammation [90]. In Drosophila models, *NIN* localizes to non-centrosomal microtubule-organizing centers, maintaining microtubule homeostasis and regulating mitochondrial function genes such as *UCP2* and *PI3K*, thereby improving metabolic stress responses [91]. This study is the first to reveal that *miR-410-3p* targets *NIN*, suggesting its involvement in muscle development and pathological processes through microtubule remodeling and inflammation regulation. Future research should further investigate the potential of *the miR-410-3p*/*NIN* axis in muscle disease treatment. *MiR-148a* is a key miRNA involved in muscle development, differentiation, and disease regulation. It is highly expressed in skeletal muscle and promotes myoblast differentiation by inhibiting *ROCK1* [92]. In chicken skeletal muscle satellite cells, it activates the PI3K/AKT pathway by targeting *DYNLL2* and *Meox2*, thereby promoting differentiation [93]. In bovine muscle cells, *miR-148a-3p* regulates proliferation and apoptosis by inhibiting *KLF6*, and it also modulates *DNMT1* and *Serpine1* in cardiac and smooth muscle, participating in cellular phenotype transitions [94]. Additionally, *miR-148a* is increasingly recognized for its potential role in skeletal muscle adipogenesis and muscle disease treatment [95]. Follistatin (*FST*) promotes muscle growth and regeneration by inhibiting myostatin (*MSTN*) and other TGF-β family members. *FST* neutralizes *MSTN* activity, increases muscle fiber cross-sectional area, activates satellite cells, and enhances *MyoD*/*Myf5* expression [96,97]. Gene therapy or recombinant protein delivery of *FST* has shown significant efficacy in treating muscle atrophy and muscular dystrophy, and it promotes myocyte proliferation through the activation of the PI3K/Akt/mTOR signaling pathway [98–100]. Optimizing *FST* structural domains (such as the N-terminal domain) can enhance efficacy and prolong drug half-life, and modified FSTL3-Fc therapy has achieved significant muscle mass improvements [101,102]. Additionally, *FST* improves insulin sensitivity and regulates fat-muscle interactions, though its effects are influenced by neural control [103–105]. This study is the first to reveal the targeting regulation of *FST* by *miR-148a*, highlighting their combined role in promoting muscle growth. The *miR-148a*/*FST* axis presents a novel target for muscle development and disease treatment. Future functional validation and mechanistic studies will provide theoretical and practical insights for livestock genetic improvement and human muscle disease therapy.

This study identified the miRNA expression changes in the skeletal muscle of Hanper sheep at different developmental stages, specifically in the comparisons of M1 vs M7, M7 vs M13, and M1 vs M13. The results suggest that specific miRNA subgroups may act as key regulatory factors at different time points of muscle growth. KEGG pathway analysis revealed regulatory mechanisms associated with miRNA expression changes at different developmental stages. Among them, the TGF-β and mTOR signaling pathways were identified as key regulators of skeletal muscle development. Numerous miRNAs modulate the TGF-β signaling pathway, which is essential for muscle development and fibrosis. For example, in this study, the differentially expressed *miR-410-3p* was reported to target *Smad7*, a negative regulator of the TGF-β signaling pathway [86]. By inhibiting *Smad7*, *miR-410-3p* may enhance TGF-β signaling, thereby promoting the proliferation and differentiation of myoblasts. Additionally, the down-regulation of the *miR-29* family, which has been shown to inhibit TGF-β-induced fibrosis, suggests its potential role in regulating extracellular matrix (*ECM*) remodeling during muscle growth. As a core regulator of protein synthesis and muscle hypertrophy, the mTOR signaling pathway regulates skeletal muscle mass by integrating growth signals and amino acid availability, which was also enriched in our analysis. This study identified *oar-miR-30a-5p* as a key regulatory factor in this pathway. In osteoarthritic chondrocytes, *miR-30a-5p* negatively regulates Akt expression, thereby inhibiting the phosphorylation of its downstream targets, p-Akt, IkB-α, and Nuclear Factor-kappa B. Since Akt is a key upstream activator of mTOR, *miR-30a-5p* may indirectly downregulate mTOR activity by suppressing Akt, thereby affecting muscle protein synthesis or metabolism [106]. Furthermore, the interaction between *oar-miR-191* and *RPS18* may play a role in ribosome biogenesis, which is crucial for efficient protein translation and muscle growth. The ribosome pathway was significantly enriched in all three comparison groups (M1 vs M7, M7 vs M13, and M1 vs M13), highlighting the importance of translation regulation in muscle development. Ribosomes are large molecular machines responsible for protein synthesis, a process essential for muscle fiber growth and repair [107,108]. The miRNA-mRNA interaction pair *oar-miR-191*/*RPS18* may influence skeletal muscle maturation by regulating ribosome biogenesis. This finding suggests a synergistic relationship between miRNA regulation and ribosome biosynthesis, enabling muscle cells to adapt to different developmental signals through precise control of protein production. This study, through comprehensive analysis, identified key miRNAs affecting muscle growth and explored their roles in the TGF-β and mTOR signaling pathways as well as ribosome biogenesis. However, further functional studies are needed through experiments, such as gene knockout or overexpression.

Although this study provides valuable insights into the miRNA regulatory mechanisms in the muscle development of Hanper sheep, there are several limitations that need to be addressed in future research. One major limitation is the small sample size, which may reduce the generalizability of the findings. While three sheep were selected at each developmental stage, increasing the sample size and diversity would help to validate the identified miRNAs and their regulatory roles, expanding the findings to a broader population. Additionally, this study only used male sheep, which may introduce gender bias, as male and female sheep may differ in muscle growth patterns and hormonal influences. Future studies should consider including both male and female sheep to investigate gender-related differences in miRNA expression and their potential effects on muscle development. Another limitation is the lack of functional validation experiments to confirm the biological roles of the identified miRNAs. Although bioinformatics tools were used in this study to predict target genes and signaling pathways, experimental validation using techniques such as luciferase reporter assays, gene silencing, or overexpression experiments is a necessary step to confirm the specific regulatory mechanisms. Functional investigations conducted both in vitro and in vivo can also clarify how these miRNAs affect muscle development, differentiation, and hypertrophy.

## Conclusions

With month-old Hanper sheep, we saw a notable increase in muscle fiber area. A few important DEMs and miRNAs were found. The pathway linked to muscle growth and development was where the target genes for the discovered DEMs were most abundant. Four new miRNAs that had not been previously discovered in sheep were among the 116 differentially expressed miRNAs that were found. These miRNAs have been linked to important pathways that control muscle growth and development, including TGF-β, mTOR, Wnt, and MAPK. Three novel miRNA-mRNA pairs (*oar-miR-133*/*MSC*, *oar-miR-148a*/*FST* and *oar-miR-410-3p*/*NIN*.) were identified as potentially playing critical roles in muscle development. This study offers insights into the crucial regulatory factors, including specific miRNAs and their associated pathways, that influence muscle fiber formation at the initial stages of Hanper sheep’s development. Furthermore, this finding will be potential critical for meat quality improvement in Hanper sheep breeding.

## Supporting information

S1 Table 116 DEMs were identified among three different groups. Sheet 1 shows 92 differentially expressed miRNAs between Hanper sheep longissimus dorsi M1 and M7 groups. Sheet 2 shows 112 differentially expressed miRNAs between Hanper sheep longissimus dorsi M1 and M13 groups. Sheet 3 shows 2 differentially expressed miRNAs between Hanper sheep longissimus dorsi M7 and M13 groups. Sheet 4 shows 116 DEMs detected in total among the three groups.
(XLSX)

S2 Table 3970 target genes of differentially expressed miRNAs at different growth stages of Hanper sheep. Sheet 1 shows the gene ID and gene name of 3970 target genes of 105 miRNAs predicted to be target genes in 116 DEMs. Sheet 2 shows the 11 miRNAs that were not predicted to be target genes in 116 DEMs. Sheet 3 shows the correspondence between 105 miRNAs predicted to be target genes in 116 DEMs and their 3970 target genes and gene descriptions.
(XLSX)

S3 Table The top15 DEMs included 22 miRNAs. Sheet 1 shows the top 15 differentially expressed miRNAs in the three groups of M1vsM7, M7vsM13, and M13vsM1, including 22 miRNAs after deduplication. Sheet 2 shows the top 15 differentially expressed miRNAs in the three groups of M1vsM7, M7vsM13, and M13vsM1.
(XLSX)

S4 Table GO enrichment of differentially expressed miRNA target genes. Sheet 1 shows the GO enrichment of differentially expressed miRNA target genes between M1 and M7 groups. Sheet 2 shows the GO enrichment of differentially expressed miRNA target genes between M1 and M13 groups. Sheet 3 shows the GO enrichment of differentially expressed miRNA target genes between M7 and M13 groups.
(XLSX)

S5 Table KEGG enrichment analysis of differentially expressed miRNA target genes. Sheet 1 is the KEGG enrichment pathway after removing duplicates of the target genes of the three groups of differentially expressed miRNAs: M1vsM13, M7vsM13, and M1vsM7. Sheet 2 is the KEGG enrichment of the target genes of the differentially expressed miRNAs of the M1 and M13 groups. Sheet 3 is the KEGG enrichment of the target genes of the differentially expressed miRNAs of the M7 and M13 groups. Sheet 4 is the KEGG enrichment of the target genes of the differentially expressed miRNAs of the M1 and M7 groups. The full protocol has been published on protocols.io and can be accessed via: https://doi.org/10.17504/protocols.io.5qpvoox5xv4o/v1.
(XLSX)
